# Preparation and Characterization for the Thermal Stability and Mechanical Property of PLA and PLA/CF Samples Built by FFF Approach

**DOI:** 10.3390/ma16145023

**Published:** 2023-07-16

**Authors:** Mengyu Cao, Tianqi Cui, Yuhang Yue, Chaoyu Li, Xue Guo, Xin Jia, Baojin Wang

**Affiliations:** 1 College of Mechanical Science and Engineering, Northeast Petroleum University, Daqing 163318, China; cmy_gx@nepu.edu.cn (M.C.); 13630321698@163.com (T.C.); 15533140532@163.com (Y.Y.); gx_cmy@nepu.edu.cn (X.G.); bjwangbaojin@nepu.edu.cn (B.W.); 2 Procurement & Equipment Department, China National Petroleum Corporation, Beijing 100007, China; jiaxin01@cnpc.com.cn

**Keywords:** fused filament fabrication approach, polylactic acid, carbon fiber, thermal stability, mechanical property

## Abstract

Currently, the mechanical performances of polylactic acid (PLA) samples prepared using the fused filament fabrication (FFF) technique are relatively poor. Hence, the carbon fiber (CF) is used to improve the thermal stability and mechanical property of FFF-ed PLA samples in this paper. The crystalline structure, thermal stability, melt flow rate, tensile strength and fractured surface morphology of PLA and PLA/CF samples were investigated with an X-ray diffraction device, differential scanning calorimeter, thermogravimetric analyzer, melt flow rate equipment, universal tensile test machine and scanning electron microscope, respectively. Meanwhile, the reinforcement mechanism of CF on the mechanical property of PLA samples was also analyzed. XRD results revealed that the diffraction peaks intensities of PLA/CF sample were obviously lower than those of PLA sample. TGA and DSC curves illustrated that the initial thermal decomposition temperature, thermal stability and crystallinity of the PLA/CF sample improved significantly. The tensile strength of the PLA/CF sample was 91.58 MPa, which was 42.49% higher than that of the PLA sample. Moreover, SEM images showed that the fractured behavior of the PLA sample varied from brittle fracture to ductile fracture after the introduction of CF. The results concluded the CF is a feasible fiber for enhancing the performances of the PLA sample.

## 1. Introduction

Fused filament Fabrication (FFF), which had high commercialization among multitudinous additive manufacturing techniques, is a popularly used approach for preparing complex-shaped thermoplastic components [1]. During the FFF process, the product is designed by 3D software and established in the manner of accumulative layers [2]. Without other auxiliary implements, the 3D digital model is directly turned into a complete product via the FFF method. In addition, the FFF approach over traditional manufacturing method possesses obvious advantages such as high automation and utilization rate of material, design degrees of freedom, easy cleaning maintenance and simple production process [3]. Therefore, the FFF technique is widely applied in the fields of automobile, aerospace, education, medicine, and mechanical engineering [4].

Compared with other materials, the high-molecular polymer (i.e., acrylonitrile butadiene styrene, polycarbonate, polyamide and polylactic acid), because of its light weight, easy processing and good mechanical property, is becoming a hotspot of the current research [5]. Among numerous polymers, the polylactic acid (PLA) is eco-friendly, cheap and non-toxic, which is an ideal filament material for fabricating FFF-ed samples [6]. However, the toughness and strength of PLA parts prepared using the FFF method are relatively bad [7]. To improve the toughness and strength, some scholars study the relationship between operation parameters and mechanical properties of FFF-ed PLA components. For example, Rajpurohit et al. [8] studied the effect of process parameters on the flexural strength of FFF-ed PLA parts. They found that the flexural strength of FFF-ed PLA parts was principally influenced by the height of layer followed by the raster angle. Lee et al. [9] discussed the various cooling air velocities on the mechanical strength and dimensional quality of a FFF-ed PLA specimen. The result indicated that the cooling air speed of 5 m/s was four times higher than 0 m/s on the tensile strength of the FFF-ed PLA part. Behzadnasab et al. [10] researched the influence of nozzle temperature impacted on the mechanical properties of the PLA part fabricated by FFF approach. They proposed that the suitable nozzle temperature contributed to the improvement of tensile strength of the FFF-ed PLA part. Dong, J et al. [11] prepared a printed polylactic acid composite material grafted with cellulose nanofibers using the 3D printing method and analyzed the effect of nanofiber and post fabrication annexing treatment on composite flexible properties. The research results confirmed the synergistic effect of PLA g-CNFs and annealing treatment on the bending performance enhancement of 3D-printed PLA composite materials. Malagutti et al. [12] proposed a post-processing method to improve the mechanical properties of fused 3D printed parts. They prepared green composite materials using wood fiber-filled polylactic acid and polyhydroxyalkanoate and evaluated the treatment effect using density, tensile mechanical properties, and microscopic observation. The research results indicated that the subsequent processing method can greatly improve the isotropic trend of the material.

Furthermore, there are the micro or nano sizes of reinforcement phases such as organic or inorganic fibers and particles, which are also proven to be profitable for enhancing the mechanical performances of FFF-ed PLA parts [13,14]. For instance, Ghiban et al. [15] studied the different reinforced phases such as copper, aluminum and graphene on the mechanical performance of FFF-ed PLA components. Additionally, they proposed a new approach for evaluating the fractured surface of FFF-ed PLA component. Butt et al. [16] measured the tear resistance, water absorption, hardness, tensile and flexural strength of FFF-ed Cu/PLA composites. The mechanical properties of the copper-infused PLA component were much higher than that of pure PLA parts. Among these reinforced phases, carbon fiber (CF) emerges as the ideal reinforcement phase because of its high mechanic strength, light weight and low expansion coefficient, which could distinctly enhance the mechanical properties of FFF-ed PLA samples [17]. For example, Li et al. [18] explored the influence of CF on the mechanical behavior of the FFF-ed PLA part. They found that the flexural strength and tensile strength of the PLA/CF specimen were much larger than that of the pure PLA component. Tian et al. [19] investigated the continuous carbon fiber (CCF) on the mechanical performances of the FFF-ed PLA sample, and the form mechanism of various interfaces was also analyzed. They concluded the flexural strength and modulus of the PLA/CCF (27%) sample were up to 335 MPa and 30 GPa, respectively.

The majority of extant reports focus on the improvement of operation parameters on the mechanical performances of FFF-ed PLA composites, including the tensile strength, flexural strength and impact strength. However, the research about CF impacted on the thermal stability and mechanical performance of FFF-ed PLA sample was little. In this article, the PLA and PLA/CF samples were both built by FFF approach. Additionally, the effect of CF on the structure of FFF-ed PLA part was examined through X-ray diffraction. Moreover, the thermal stability of FFF-ed samples was measured with a thermal gravimetric analyzer and differential scanning calorimeter, respectively. Furthermore, the tensile strengths of the PLA and PLA/CF samples were tested with the universal testing machine. The fractured morphology of the PLA and PLA/CF samples was detected with a scanning electron microscope. The reinforcement mechanism of CF on the tensile strength of FFF-ed PLA specimen was also analyzed.

## 2. Experiment and Method

### 2.1. Preparation

**Figure 1:**
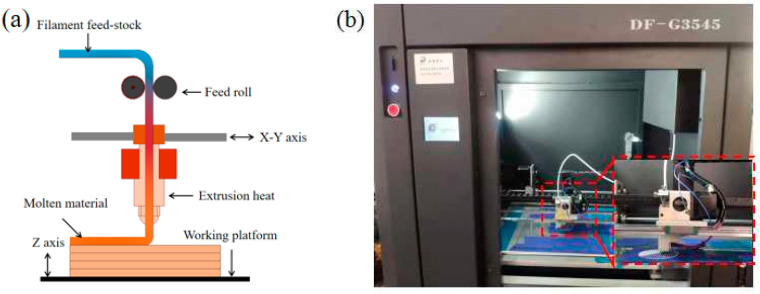
The production process of PLA and PLA/CF samples: (**a**) Schematic and (**b**) Equipment.

In this paper, the PLA granules and short CFs were both purchased from Dongguan ANT Plastic Technology Co., Ltd. (Dongguan, China). The dimension and diameter of CF were 40–60 μm and 15–20 μm, respectively. Prior to the blending process, the short CFs were modified with 20 wt.% H_2_SO_4_ solution for decreasing the surface energy. Moreover, the KH570 style silane coupling agent was used to improve the performances of PLA filament and PLA/CF filament. The PLA filament and PLA/CF filament were manufactured by a TY-7004 type single screw extrusion equipment at the extrusion temperature of 180 °C and screw speed of 200 r/min. The CF content of the PLA/CF filament was 15 wt.%. The diameters of PLA filament and the PLA/CF filament were both 1.75 mm. Before printing, the PLA and PLA/CF filaments were desiccated in the NJ101-5 type drying box at the temperature of 60 °C and kept for 50 min. The PLA and PLA/CF samples were built using a DF-G3545 type 3D printer. The production process of PLA and PLA samples is presented in Figure 1. The 3D models of FFF-ed samples were built using CAD software and were translated to machine language using Cura 15 slice software. Then, the FFF style printer was employed to establish the entity object. The filaments were heated to the molten state and extruded. Finally, the molten materials shaped and hardened on the platform. The platform temperature was set as 50 °C for reducing the residual stress. During the FFF process, the material specie was the only variable. According to our experiences, the suitable technological parameters for manufacturing PLA and PLA/CF samples are listed in Table 1.

### 2.2. Characterization

The X-ray diffraction (XRD, TD-3700) device was used to detect the structure of FFF-ed samples. The operation condition of XRD device was set to Cu Kα (λ = 1.54 nm), 2 kV, and the scanning scope (2θ) from 10° to 60° at a scanning rate of 0.02°/s.

According to the standard of ASTM D6370, the thermogravimetric analyzer (TGA, Netzsch STA449C) was applied to investigate the component of FFF-ed samples. Under the nitrogen atmosphere, the 10 mg of FFF-ed samples was placed in the platinum plate and heated from 25 °C to 800 °C at a rate of 10 °C/min.

The differential scanning calorimeter (DSC, HY4510) was used to measure the crystallization and melting behavior of PLA and PLA/CF samples. The FFF-ed PLA and PLA/CF samples in the atmosphere of N_2_ gas were firstly heated from room temperature to 210 °C, at a rate of 20 °C/min. The sample was kept at 210 °C for 3 min, and the object was to eliminate thermal history. Then, the sample was cooled to 30 °C at a rate of 10 °C /min and heated to 200 °C at the same rate. Finally, the DSC curves of sample were recorded and analyzed. The methods used in this part of the work have also been used in the work of Hsieh et al. [20] and Zhu et al. [21]. The crystallinity ($\chi_{c}$) of PLA and PLA-CF samples was calculated using the following equation:

$$\chi_{c}=\frac{{∆H}_{m}-{∆H}_{c}}{\omega \times ∆H_{100}}$$

where ${∆H}_{m}$ represents the melting enthalpy; ${∆H}_{c}$ acts as enthalpy of cold crystallization, ω serves as the mass fraction of PLA in the sample, and $∆H_{100}$ is the standard enthalpy of PLA forms fully crystallized, which reads as 93.0 J/g [22].

According to the standard of GB/T3682, the melt flow rates (MFR) of PLA and PLA/CF samples were inspected using a HY4310B style MFR tester. The test temperatures of FFF-ed samples were configured to 210 °C. The MFR data of PLA components tested under the standard load of 2.16 kg and shearing time of 10 s, which was recorded for 3 times and averaged.

**Figure 2:**
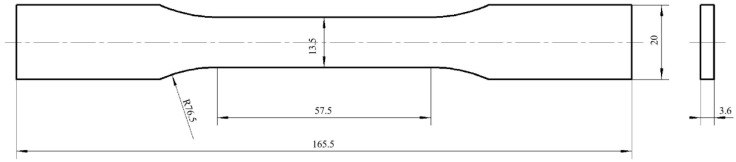
Specific dimension of PLA and PLA/CF samples prepared through the FFF method in the tensile test (unit: mm).

**Figure 3:**
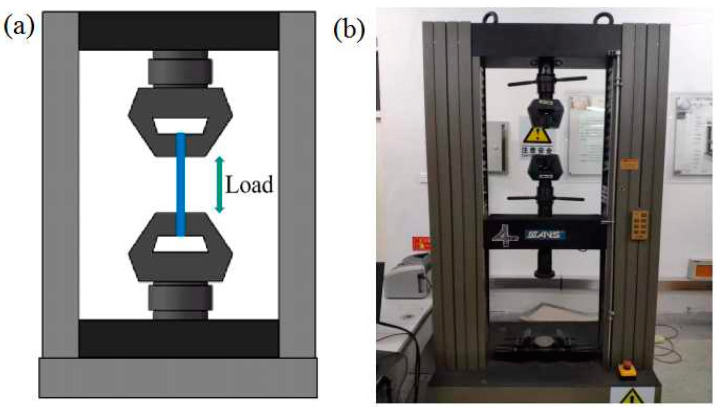
Schematic and equipment of the tensile test: (**a**) schematic and (**b**) equipment.

Figure 2 shows the specific size of PLA and PLA samples produced using the FFF method in the tensile test. The FFF-ed samples were conducted in the tensile test according to the standard of ASTM D638-14. Figure 3 [23] indicates the schematic and experimental installation of universal tensile test machine, which was produced by Changchun Haoyuan Company, with a measuring range of 10 kN. The FFF-ed samples were stretched until fracture under the working conditions of static load and the cross-head at a speed of 5 mm/min. The measurement data were recorded using a CREE-8003A style universal tensile test machine for 5 times and averaged. The fractured surfaces of samples received the treatment of gold spray. The fractured surface morphology of FFF-produced samples were investigated using a scanning electron microscope (SEM, FEG450).

## 3. Results and Discussion

### 3.1. XRD Pattern Observation

**Figure 4:**
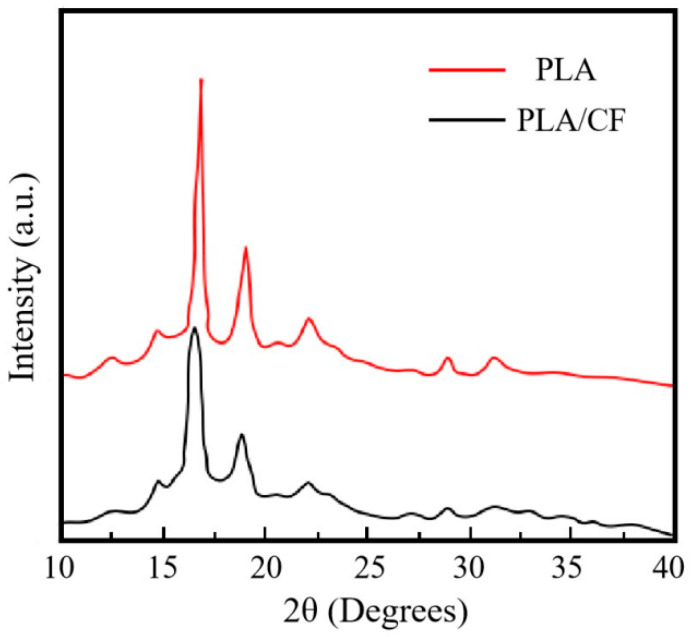
XRD patterns of PLA and PLA/CF samples produced by FFF method.

Figure 4 reveals XRD patterns of the PLA and PLA/CF samples obtained by FFF approach. The diffraction peaks locations of the PLA/CF sample were same as those of the PLA sample, which illustrated the absent influence of CFs on the crystal type of PLA sample [24]. In addition, the new phase was not generated in the PLA/CF sample. This result meant that the chemical reaction between CF and PLA had not happened, which was similar to the investigation proposed by Abu-Jdayil et al. [25]. The diffraction peaks of the PLA and PLA/CF samples both emerged at 16.6° and 19.1°, which corresponded to the crystal plane of (200/110) and (203), respectively [26]. The diffraction peaks of the PLA/CF sample had lower intensity and were broader than those of the PLA sample, which contributed to the heterogeneous nucleation of CF which refined the crystal size of PLA sample. This result was proved by the research of Li et al. [27]. Furthermore, the diffraction peaks of CF corresponding to 16° and 19.18° were not discovered in Figure 3. It was because the diffraction peak intensity of CF was covered by that of PLA, which was confirmed by the report of Lei et al. [28].

### 3.2. Thermal Stability Analysis

**Figure 5:**
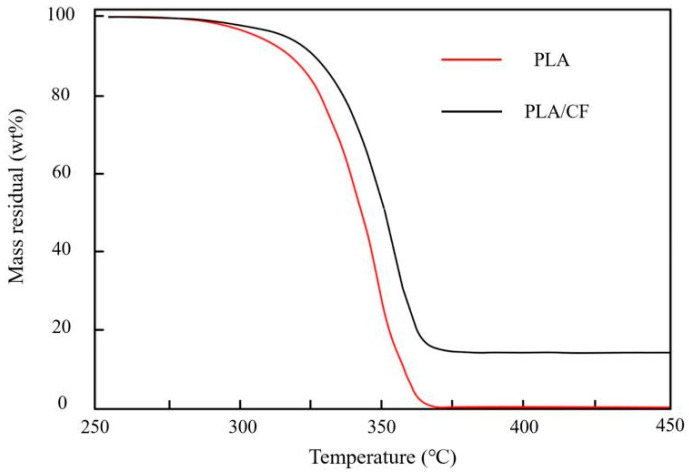
TGA curves of PLA and PLA/CF samples fabricated by FFF approach.

Figure 5 presents TGA curves of the PLA and PLA/CF samples fabricated using the FFF approach. The initial thermal decomposition temperature (T_b_) and residual rate of FFF-ed samples from TGA curves are shown in Table 2. It could be seen that the PLA material of the FFF-ed samples could be absolutely decomposed after temperature exceeded 375 °C. Furthermore, the T_b_ of PLA and PLA/CF were 280.6 °C and 291.8 °C, respectively. Compared with the PLA sample, the T_b_ of PLA/CF sample had significant growth. The reasons of this phenomenon could be explained as follows: (1) The thermal conductivity of CF was much higher that of PLA, which was also proven by the report of Ye et al. [29] who proposed that the thermal conductivity of polymer was lower than that of carbon fiber in the thermal path theory. The heat could be rapidly transferred from a local region to the whole region and uniformly distributed in the PLA/CF sample, which led to the T_b_ of PLA/CF sample increasing obviously. The result was similar to the research of Klaser et al. [30] about the glass-fiber (GF) on the thermal stability of the PLA specimen. (2) The surface of CFs was coated with a molecular chain of PLA and generated network structure similar to cross-linked compounds in the different crystal interweaves, resulting in the T_b_ of PLA/CF sample being higher than that of PLA sample. (3) The uniform distribution of CFs in the PLA/CF sample acted as the function of heterogeneous nucleation, which had obvious influence on the crystallization improvement of the PLA/CF sample. Siengchin et al. [31] and Adomaviciute et al. [32] demonstrated that the heterogeneous nucleation of fiber was conducive to the crystallization growth of PLA-printed parts. Therefore, the addition of CF is an effective method to improve the thermal stability of the PLA sample.

**Figure 6:**
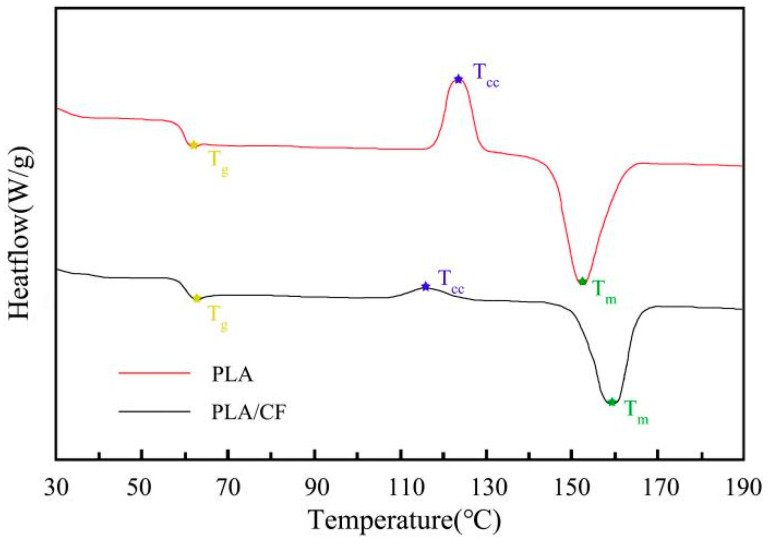
DSC curves of PLA and PLA/CF samples obtained by FFF approach.

Figure 6 displays the DSC curves of PLA and PLA/CF samples prepared using the FFF method. The glass transition temperature (T_g_), cold crystallization temperature (T_cc_) and melt temperature (T_m_) of FFF-ed samples are listed in Table 3. The T_g_ of the PLA sample and PLA/CF sample were 61.32 °C and 62.13 °C, respectively. The result indicated that the introduction of CF had little influence on the T_b_ of the FFF-ed PLA sample. By contrast, after the introduction of CF, the T_cc_ and T_m_ of the PLA sample decreased from 124.75 °C to 115.63 °C and increased from 151.91 °C to 159.87 °C, respectively. The result demonstrated that the introduction of CF had significant influence on the T_c_ and T_m_ of the FFF-ed PLA sample. Furthermore, the incorporation of CF resulted in the cold crystallization peak of the FFF-ed PLA sample being significantly moved to left, which meant that the FFF-ed PLA/CF sample was easy to crystallize. The reason was attributed to the heterogeneous nucleation dominating the crystallization process after the incorporation of CF, which impacted the crystallization rate much more than the crystallization mechanism of the PLA sample [33]. The result was confirmed through the XRD pattern in Figure 4. In general, the high X_c_ of the FFF-ed sample was beneficial to improving the thermal stability [34]. Therefore, the thermal stability of the PLA/CF sample is stronger than that of the PLA sample.

### 3.3. MFR Investigation

**Figure 7:**
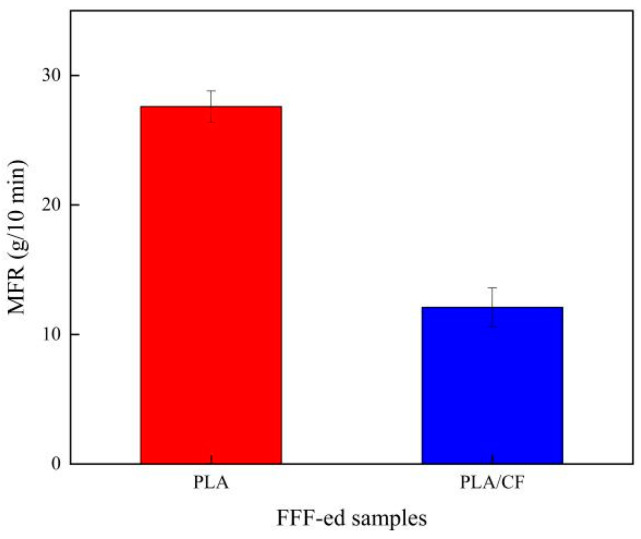
MFR of PLA and PLA/CF filaments fabricated by FFF technique.

Figure 7 illustrates the MFR of PLA and PLA/CF samples produced using the FFF method. The MFR of PLA sample was 27.6 g/10 min, while that of the PLA/CF sample was only 12.1 g/10 min. The reasons of this result could be illustrated as follows: (1) The reinforced phase of CF existed in the PLA sample and caused the movement obstruction of the PLA molecular chain and the increase of internal friction, resulting in the MFR of the PLA/CF sample decreasing [35]. (2) In addition, the melt temperature and crystallization degree of PLA/CF sample were higher than those of PLA sample, which was proven through the DSC curves in Figure 6. The high melt temperature and crystallization degree led to the MFR of the PLA/CF sample decreasing [36]. Therefore, these reasons caused the MFR of PLA sample being larger than that of the PLA/CF sample.

### 3.4. Mechanical Property Measurement

**Figure 8:**
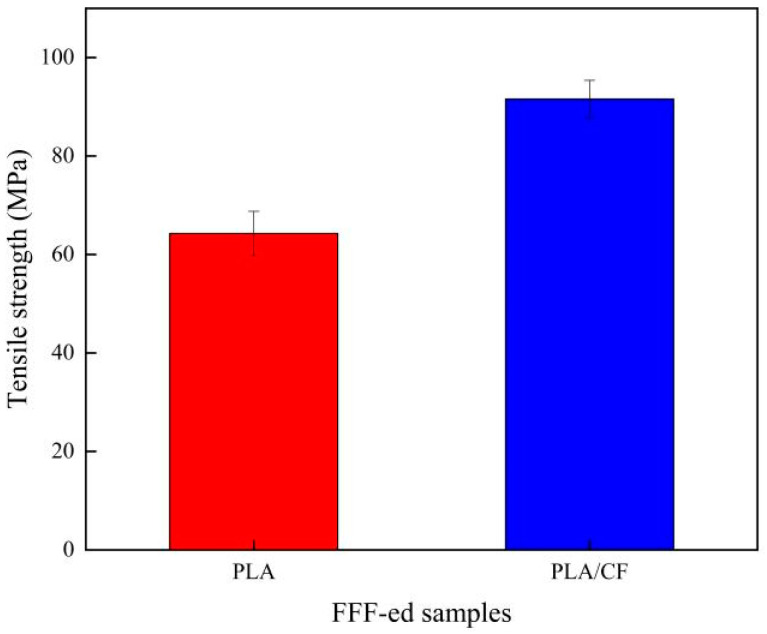
Tensile strength of PLA and PLA/CF samples obtained by FFF method.

Figure 8 shows the tensile strength of the PLA and PLA/CF samples manufactured using the FFF technique. The tensile strengths of the PLA and PLA samples were 64.27 MPa and 91.58 MPa, respectively. After the CF was introduced, the tensile strength of PLA sample increased obviously. The reason for this phenomenon could be illustrated as the CF of the PLA/CF sample serving as the action of reinforcing frame structure and heterogeneous nucleation. On the one hand, the tensile strength of CF was up to 4000 MPa, which could enhance the tensile strength of the PLA/CF sample distinctly. On the other hand, the heterogeneous nucleation of CF contributed to the growth of the crystallization degree, resulting in the tensile strength of the PLA/CF sample increasing. This result is similar to the study of Zhu et al. [37] about heterogeneous nucleation for enhancing the tensile strength of poly(butylene succinate)/PLA composites. The internal mechanism of its stress deformation can be further explained through the mechanism model established by Kumar Mishra et al. [38]. In addition, the uniform dispersion of CFs in the PLA could have effectively transferred and absorbed the external load, which further enhanced the tensile strength. This outcome was proven by the research of Qian et al. [39] about the function of CF in the PLA-thermoplastic poly(ether)urethane composites and the research of Malagutti et al. [40] about Tensile properties of FDM 3D-printed wood flour-filled polymers and mathematical modeling through classical lamination theory.

### 3.5. Fractured Surface Morphology Detection

**Figure 9:**
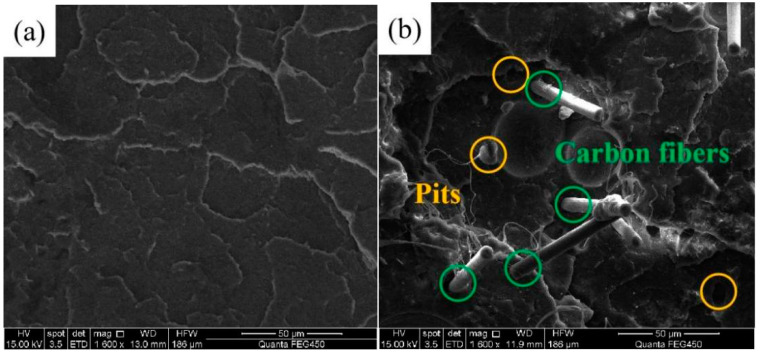
Fractured surface morphology of (**a**) PLA and (**b**) PLA/CF samples manufactured through the FFF approach.

Figure 9 exhibits the SEM photographs of the fractured surface morphology of the PLA and PLA/CF samples built with FFF technology. Figure 9a shows the fractured surface morphology of the PLA sample presenting relatively smooth. By comparison, the fractured surface morphology of the PLA/CF sample appeared rough and uneven (seen in Figure 9b). SEM images indicated that the fractured behavior of PLA sample varied from brittle fracture to ductile fracture after the introduction of CF, which was similar to the research proposed by Raj et al. [41]. Furthermore, some obvious pits and damaged CFs existed in the fractured surface of the PLA/CF sample. The result is attributed to the fine adhesion force between the CFs and PLA matrix. The minor pits were produced from the stretch of CFs in the PLA matrix during the tensile test. In addition, the carbon fibers distributed in the PLA/CF sample pointed to the same orientation, which indicated that the suitable layer thickness contributed to the uniform arrangement of CFs orientation [42].

### 3.6. Reinforcement Mechanism

**Figure 10:**
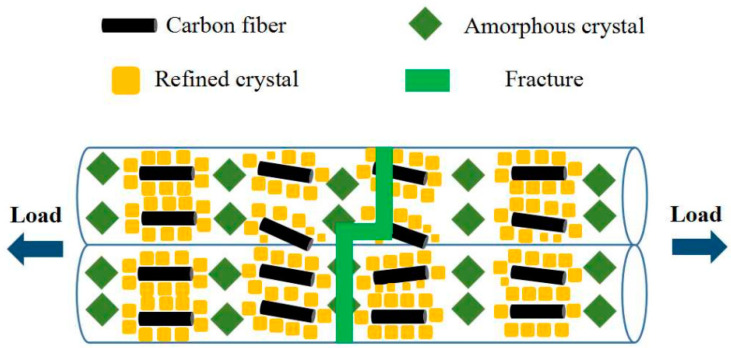
Reinforcement mechanism of carbon fiber on the mechanical property of PLA.

Figure 10 demonstrates the reinforcement mechanism of carbon fiber on the properties of the PLA sample. The low MFR of the PLA/CF sample caused the increased adhesion force between filaments, which resulted in the mechanical property of the PLA/CF sample improving [43]. Meanwhile, the same orientation of CFs in the PLA/CF sample could effectively transfer and absorb the external load, which could significantly improve the mechanical property [44]. Moreover, the uniform distribution of carbon fibers dispersed in the PLA/CF sample acted as the heterogeneous nucleation and decreased the crystallization temperature, which increased the degree of crystallization and enhanced the mechanical property of PLA/CF sample [45].

## 4. Conclusions

(1) XRD results showed that the diffraction peaks of the PLA and PLA/CF samples were located at 16.6°and 19.1°, corresponding to the crystal planes of (200/110) and (203), respectively. After the introduction of carbon fiber, the crystalline size of PLA was refined.
(2) TGA and DSC curves illustrated that the thermal stability and crystallization degree of the PLA/CF sample were better than those of the PLA sample. In addition, the MFR of the PLA sample decreased from 27.6 g/10 min to 12.1 g/10 min after CF was introduced.
(3) Compared with the PLA sample, the tensile strength of PLA/CF sample increased from 64.27 MPa to 91.58 MPa. SEM images showed that the fractured behavior of the PLA sample varied from brittle fracture to ductile fracture after the CF was introduced.

## Figures and Tables

**Table 1:** Technological parameters for manufacturing PLA and PLA/CF samples.

Parameters	Specific
Printing speed (mm/s)	60
Nozzle diameter (mm)	0.4
Layer thickness (mm)	0.2
Supplied speed (mm/s)	60
Raster angle (°)	0
Print temperature (°C)	200
Infilled rate	100%

**Table 2:** Specific data from TGA of PLA and PLA/CF samples manufactured by FFF technology.

Sample	T_b_	Residual Rate
PLA	285.6 °C	0.74%
PLA/CF	291.8 °C	15.76%

**Table 3:** Thermal properties of PLA and PLA-CF samples prepared by FFF method.

Specimen	T_g_ (°C)	T_cc_ (°C)	T_m_ (°C)	${∆H}_{m}$ (J/g^−1^)	${∆H}_{c}$ (J/g^−1^)	X_c_ (%)
PLA	61.32	123.68	151.91	57.3	45.2	13.01
PLA/CF	62.13	115.63	159.87	54.6	9.5	48.49

## Data Availability

Data available on request due to restrictions eg privacy or ethical. The data presented in this study are available on request from the corresponding author. The data are not publicly available due to security.
